# Supplementary material for: Efficacy of Modified Treat-and-Extend Aflibercept Regimen for Macular Edema Due to Branch Retinal Vein Occlusion: 1-Year Prospective Study
Source: J Clin Med. 2020 Jul 23;9(8):2360. doi: 10.3390/jcm9082360 (PMC7464624; doi:10.3390/jcm9082360)
Supplement: Supplementary file 1 [file jcm-09-02360-s001.zip › Supplement.docx]

**Efficacy of modified treat-and-extend aflibercept regimen for macular edema from branch retinal vein occlusion: 1-year prospective study**

Yusuke Arai^1^, Hidenori Takahashi^1,2,3,*^, Satoru Inoda^1^, Shinichi Sakamoto^1^, Xue Tan^2,3^, Yuji Inoue^1,3^, Satoko Tominaga^1^, Hidetoshi Kawashima^1^, Yasuo Yanagi^5,6,7^

^1^ Department of Ophthalmology, Jichi Medical University, Shimotsuke, Japan

^2^ Japan Community Health Care Organization Tokyo Shinjuku Medical Center, Tokyo, Japan

^3^ Department of Ophthalmology, Graduate School of Medicine, University of Tokyo, Tokyo, Japan

^4^ Department of Ophthalmology, Asahikawa Medical University, Asahikawa, Japan

^5^ Medical Retina, Singapore National Eye Centre, Singapore, Singapore

^6^ Medical Retina, Singapore Eye Research Institute, Singapore, Singapore

^7^ The Ophthalmology & Visual Sciences Academic Clinical Program, Duke-NUS Medical School, National University of Singapore, Singapore

***Corresponding author**: Hidenori Takahashi

Department of Ophthalmology, Jichi Medical University

3311-1 Yakushiji, Shimotsuke-shi, Tochigi 329-0431, Japan

Tel.: +81- 285-58-7382; Fax: +81- 285-44-8365

E-mail: takahah-tky@umin.ac.jp

**Supplementary information**

Supplementary Table S1 lists the fundus cameras and OCT devices of each institution.

**Supplementary Table S1: Fundus cameras and OCT devices of each institution**

Table S1. Fundus camera and OCT of each institution

| Institution | Camera | OCT |
| --- | --- | --- |
| A | VX-10 | RS-3000, DRI OCT Toriton |
| B | TRC-50DX Type IA | Cirrus HD-OCT Model 4000 |
| C | VX-10i | Cirrus HD-OCT Model 4000 |
| D | VX-10 | Cirrus HD-OCT Model 4000 |
| E | VX-10 | RS-3000 |
| F | Optos200Tx | RS-3000 |

Institution A: Jichi Medical University Hospital, B: Japan Community Health Care Organization Tokyo Shinjuku Medical Center, C: Ohkubo Eye Clinic,

D: Takahashi Eye Clinic E: Saito Eye Clinic, F: Aoki Eye Clinic.

VX10, VX-10i: Kowa Co Ltd., Tokyo, Japan

TRC-50DX Type 1A, DRI OCT Toriton; Topcon Medical Systems, Tokyo, Japan

Optos200Tx: Optos PLC, Dunfermline, UK

RS-3000: Nidek Co., Ltd., Tokyo, Japan

HD-OCT Model 4000; Carl Zeiss Meditec, Jena, Germany

Abbreviation: OCT, optical coherence tomography.
